# The Way of Research

**Published:** 1921-03-05

**Authors:** 


### THE WAY OF RESEARCH.

### BY A CORRESPONDENT.

There is surely no reason why historical methods should not be applied when we inquire as to how modern medical research may be best promoted. And among the things the historical method reveals is a familiar progress from the homogeneous to the heterogeneous. Bright and Addison and Basedow were general physicians, who, by keen perception and brilliant induction, separated out diseases for our recognition. Pasteur was a giant. But the greatest application of his biological work—that to surgery—was made by a surgeon. Koch and Sir Patrick Manson both became almost specialists, one in tuberculosis, the other in tropical medicine. In really "path-breaking" work Horsley was a neurologist pure and simple. Sir Ronald Ross gave up many years of his life to the study of malaria. So far, then, it is a case, if results count, of the cobbler sticking to his last, of exclusive devotion to one disease or one subject. Of course, there are instances to the contrary which can be put forward by objectors. The examples of the great Ehrlich, of the great Helmholtz, and—less in degree, but similar in kind—of Sir Almroth Wright, show that a generalised investigatory activity may be highly fruitful too. But this latter is plainly the rarer case.

### The Future in this Country.

These brief considerations have a strong bearing upon current expectations and appointments. Many educational authorities are now naturally wishful that this country should make up its leeway in notable original work. It is interesting to see how far their plans tally with the lessons of the past. With one lesson of the past, that just given above, it is plain they do not tally. Take, for instance, the unit system of medical education, which has "made good," as one says, in face of the prejudice excited by its Teutonic origin, and—a much harder matter, seemingly—in face of its *lèse-majesté* against the British nursing system. Under this plan a general surgeon, or a general physician mostly, has beds allotted to him for the purpose of general research *cum*-education. Obviously such research—here at least, and as a routine—will be but little intensive. Take, again, the appointment of a general pathologist practically to the post of physician, as has been the case for many years at a London hospital, an example beginning to be followed in the provinces. Here a general pathological or bacteriological research worker attacks various desperate investigatory problems. Take, lastly, an extreme case we do not intend further to particularise. Here a not very distinguished general pathologist has been given wide administrative powers in a big public health undertaking, and also the direction of research on a subject in which general estimation, as expressed by public correspondence, judges him to be poorly qualified.

All this seems to those guided by history to be rather unfortunate; and for reasons not only *a priori*. The special literatures of the great diseases have long been so vast that daily familiarity with them for several years is indispensable to a proper orientation. Failing such orientation, our researcher may, in the easiest possible way, waste his time in duplication and supererogation. And since even the London medical libraries have long had to relinquish stocking every special journal, the war-time special literature of many a subject is very hard for a new hand to come by. Thus general subjects for inquiry would have to be chosen, whereas nowadays it is research on highly specialised points that is really required. Again, what clearly dictates this preference so clearly shown for the pathologist, bacteriologist, or physicist as investigators is their familiarity with technique, at least the technique of the physical laboratory. Their temperament for research may be all to seek, since we cannot get a Sir Almroth Wright every day. And temperament will always beat technique: those who ever saw Sir Patrick Manson's little microscope in its leather travelling-case will not need telling this.

### The Idea and the Experiment.

The man with the true new idea can always in time manage his verification, or get such parts of it done as he cannot manage, to the general satisfaction. Whereas the many many men with hundreds of elaborate, unoriginal experiments, but no new idea, merely encourage the printing trade. There is one point in which the salaried pathologist may have an advantage over the honorary physician, for he is nearly always much less distracted by the claims of private practice. However, there are many young physicians who would be glad to take up a subject and work at research in it on a salary, as at present pathologists do. Moreover, instances have been known of pathologists making a good deal of money in private practice.

To sum up, then, for a long time yet clinical familiarity with a disease must be the *chief* qualification for directing research therein. Of course, in such a vast undertaking as the advancement of human knowledge there must be room for all kinds of researches; but the current tendency to exalt the handicraftsman over the thinker is unsound. If the thinker has to think overmuch about his money takings, his means of livelihood, to the detriment of his best work, why then he must be relieved of undue anxiety on that score. And if, as is likely, he has intellectual emulation, he will be willing enough for some sacrifice.

